# Whole-Genome Resequencing and Transcriptomic Analysis to Identify Genes Involved in Leaf-Color Diversity in Ornamental Rice Plants

**DOI:** 10.1371/journal.pone.0124071

**Published:** 2015-04-21

**Authors:** Chang-Kug Kim, Young-Joo Seol, Younhee Shin, Hye-Min Lim, Gang-Seob Lee, A-Ram Kim, Tae-Ho Lee, Jae-Hee Lee, Dong-Suk Park, Seungil Yoo, Yong-Hwan Kim, Yong-Kab Kim

**Affiliations:** 1 Genomics Division, National Academy of Agricultural Science (NAAS), Rural Development Administration (RDA), Jeonju, Korea; 2 Codes division, Insilicogen Inc., Suwon, Gyeonggi-do, Republic of Korea; 3 Biosafety Division, NAAS, RDA, Jeonju, Korea; 4 Policy Development Office, Korea Institute of Planning & Evaluation for Technology in Food, Agriculture, Forestry & Fisheries, Anyang, Korea; 5 School of Electrical Information Communication Engineering, Wonkwang University, Iksan, Korea; National Institute of Plant Genome Research, INDIA

## Abstract

Rice field art is a large-scale art form in which people design rice fields using various kinds of ornamental rice plants with different leaf colors. Leaf color-related genes play an important role in the study of chlorophyll biosynthesis, chloroplast structure and function, and anthocyanin biosynthesis. Despite the role of different metabolites in the traditional relationship between leaf and color, comprehensive color-specific metabolite studies of ornamental rice have been limited. We performed whole-genome resequencing and transcriptomic analysis of regulatory patterns and genetic diversity among different rice cultivars to discover new genetic mechanisms that promote enhanced levels of various leaf colors. We resequenced the genomes of 10 rice leaf-color accessions to an average of 40× reads depth and >95% coverage and performed 30 RNA-seq experiments using the 10 rice accessions sampled at three developmental stages. The sequencing results yielded a total of 1,814 × 106 reads and identified an average of 713,114 SNPs per rice accession. Based on our analysis of the DNA variation and gene expression, we selected 47 candidate genes. We used an integrated analysis of the whole-genome resequencing data and the RNA-seq data to divide the candidate genes into two groups: genes related to macronutrient (i.e., magnesium and sulfur) transport and genes related to flavonoid pathways, including anthocyanidin biosynthesis. We verified the candidate genes with quantitative real-time PCR using transgenic T-DNA insertion mutants. Our study demonstrates the potential of integrated screening methods combined with genetic-variation and transcriptomic data to isolate genes involved in complex biosynthetic networks and pathways.

## Introduction

The leaves of plants are evaluated by studying the major photosynthetic organ. The leaf color, size, and shape directly affect photosynthesis, crop yield, and grain quality. Previous studies of leaf color in rice focused on characterizing the correlation between fertilizer nitrogen and chlorophyll metabolism for photosynthesis. The leaf-color chart can indirectly estimate the nitrogen status of growing rice [1], and chlorophyll metabolism is very important to all plants in relation to photosynthesis [2]. In addition, the leaf color is an effective marker to identify hybridization in rice, because color phenotypes can be easily identified. Common leaf-color mutations are associated with the albino, chlorosis, thermo-color, light green, maintaining green, stripes and zebra, green-revertible albino, dark-green, and purple phenotypes. Using various leaf-color mutants, genetic mechanisms have been analyzed to reveal the functions of color-related genes involved in chlorophyll biosynthesis [3], chloroplast structure and function [4], the regulation of chloroplast development, carotenoid biosynthesis, anthocyanins biosynthesis [5,6], programmed cell death, and photosynthesis [7]. Leaf color-mutant characters are normally controlled by recessive nuclear genes, with only a few cases of control by dominant or cytoplasmic genes [8].

Rice cultivation requires an optimum nutritional balance. Variation in rice-leaf color is caused by genetic factors and the cultivation environment (i.e., deficiencies and toxicities of macronutrients and microelements). The leaf color is strongly affected by elemental macronutrient characteristics including sulfur deficiency, low amounts of magnesium, and phosphoric acid deficiency (http://www.knowledgebank.irri.org/). Elemental macronutrients are important to a plant's growth and affect the color of the leaves. Magnesium is an essential macronutrient, and low amounts of magnesium cause the main symptom of chlorosis, or yellowing between leaf veins, giving the leaves a marbled appearance [9,10]. Sulfur deficiency causes a uniform, pale-green chlorosis throughout the plant; younger leaves turn yellow first, sometimes followed by older leaves [11]. Phosphate starvation causes the leaves of rice plants to develop prominent dark-green coloration, and phosphorus deficiency is associated red and purple coloration in the leaves of rice accessions that have a tendency to produce anthocyanin [12,13].

Recently, rice field art using ornamental rice has become popular throughout China, Japan, Korea, and other regions. Rice field art is a large-scale art form in which people design rice fields using various kinds of plants with different leaf-color characteristics. No chemicals are used to color the ornamental rice fields; letters are drawn with the use of multi-colored leaves (i.e., purple, yellow, green, and red). Despite the role of various metabolites in determining the color of rice leaves, comprehensive studies of color-specific metabolites in ornamental rice have been limited. We performed whole-genome resequencing and a transcriptomic analysis to determine the patterns of genetic diversity and gene regulation to discover new genetic mechanisms that promote enhanced levels of various colors in cultivated rice.

## Materials and Methods

### Rice materials

We prepared samples of 10 different rice accessions for genomic DNA and transcriptomic analysis. Three leaf-color cultivars [*Hwangdo* (accession: IT264739), *Jado* (accession: IT210918), and *Dongjin* (accession: IT163355)] were collected from the RDA-Genebank (http://www.genebank.go.kr/). Seven leaf-color mutant lines (*D052*, *D056*, *D101*, *D120*, *D122*, *D128*, and *D131*) were derived from the *Dongjin* cultivar using the gene-trap system [14]. The 10 rice accessions show diverse coloration and patterning: *Dongjin* is green; *Hwangdo* is yellow; *Jado* is dark red; *D052* is yellowish green; *D056* is yellowish green; *D101* is mixed purple and green; *D120* is dark-brown spotted; *D122* is mixed white and green; *D128* is mixed albino and green; *D131* is yellowish zebra (S1 Fig). For the transcriptome analysis, we performed a total of 30 RNA-seq experiments that compared gene expression in the 10 accessions at three different stages of leaf development (germination + 30 days, + 45 days, and + 60 days).

### Library construction and sequencing of genomic DNA

The paired-end sequencing library with an insert size of 400 bp was constructed using the TruSeq DNA Sample Preparation Kit v2 (Illumina Inc., San Diego, CA, USA) following the manufacturer's protocols (Illumina Inc.). The whole-genome resequencing was performed using Illumina Hiseq 2000 sequencing platforms. We collected the fragments, which were sequenced for 101 cycles (101 bp), from each genomic library using the paired-end sequencing method.

### Reads mapping

We checked the quality-score distribution of each library. If a sequenced base had a quality score less than Q20, which indicates an accuracy of 99% for the base call, the base call was changed to “N”. Reads with fewer than 90 bp or with Ns at more than 10% of their total base positions were removed. The filtered reads were aligned to the reference genome MUS Rice Genome Annotation Project Release 7 (Os-Nipponbare-Reference-IRGSP-1.0) [15] using the CLC Assembly Cell software (CLC Bio, Aarhus, Denmark) with the default options (80% identity and 50% coverage by high-scoring base pairs).

### SNP detection and analysis

SNPs and small InDels were identified in the reference-assembled sequences using the “find variations” function of the CLC Assembly Cell software with the following parameters: minimum depth = 10, minimum mismatch count = 5, limit fraction ≥35%. To reduce the erroneous SNP calls caused by the uncertainty of multiple sequences mapping to high sequence-similarity regions in the reference genome, we filtered out the low-quality mapping regions that lacked 95% sequence identity and 100% coverage by high-scoring base pairs. To further characterize the SNPs, we categorized their locations as either genic or intergenic. Less than half of the SNPs were localized in genic regions; most of them were located in intronic regions. In addition, we determined whether the amino acid characteristics were changed by the non-synonymous SNPs (e.g., hydrophobic to basic or amino acid to stop codon), because compositional changes in amino acids can change the structural conformation or enzymatic activities of proteins, generating phenotypic diversity and critical functional variations.

### Structural analysis

We performed a population-structure analysis of the SNPs using the FRAPPE program, which is based on the maximum likelihood method [16]. We constructed PED files for the 10 rice accessions using 10,000 iterations and considering cluster numbers (K) from two to seven [17]. To construct the phylogenetic tree, we calculated the genetic distances between the different accessions using the SNPs. The neighbor-joining method was applied to construct the tree based on the distance matrix calculated by the PHYLIP 3.695 software [Washington University, USA; (http://evolution.genetics.washington.edu/phylip/)]. FigTree 1.4.0 (http://tree.bio.ed.ac.uk/software/figtree/) was used to render the phylogenetic tree.

### Gene identification and characterization analysis

We assembled the unmapped reads from each sample into contigs using CLC Assembly Cell. The default parameters were used, and only contigs, not scaffods, were constructed. Contigs shorter than 2 kb and redundant sequences identified by self-alignment were excluded. We conducted *de novo* gene annotation with Geneid [18] for the non-redundant, novel contigs. To annotate gene functions, we used BLASTP [19] to compare the novel candidate genes with an e-value cutoff ≤1.0 e-10 using the MUS Rice Genome Annotation Project Release 7 database. Then, we performed further functional annotation using InterProScan (InterProScan-5.2–45.0) [20]. To identify gene-loss events, we first extracted high-quality gap regions, those mapped with less than 4× reads depth and having more than 20× reads depth on each 1-kb side flanking the gap region. We then isolated lost genes: those with < 20% coverage in genic region, especially in the coding DNA sequence (CDS) region. To identify genes involved in macronutrient (i.e., magnesium, and sulfur) or anthocyanin transport, the sequences were aligned using ClustalW [21] with the default options. The aligned sequences were used with the neighbor-joining and the maximum likelihood methods for phylogenetic-tree analysis with the CLC Genomics Workbench 6.5 platform (CLC Bio, Aarhus, Denmark). To determine the similarity among the 10 rice accessions, hierarchical clustering was applied to each transporter gene using Euclidean distances, and the gene pairs were illustrated using Circos [22].

### RNA-seq transcriptome analysis

For the RNA-seq experiments, library construction was performed using the general protocol [23], and sequencing and assembly were performed using the Illumina Hi-Seq 2000 platform (Hayward, CA, USA). To perform quality-control checks on the raw sequence data, we used the FastQC program (http://www.bioinformatics.babraham.ac.uk/projects/fastqc/). The reads were mapped using the Tophat software, and gene-expression levels were identified using Cufflinks with the default parameters [24]. The fragments per kilobase of transcript per million mapped reads (FPKM) score was calculated with the transcribed fragments, and functional associations were computed using the Gene Set Enrichment Analysis (GSEA) method with the normalized FPKM and Gene Ontology (GO; http://www.ebi.ac.uk/QuickGO/) categories.

### Extraction of total RNA and quantitative real-time PCR analysis

Total RNA was isolated from the leaves of the rice plants using an RNeasy plant mini kit (Qiagen, Inc., Valencia, CA, USA). For quantitative real-time PCR (qPCR), first-strand cDNA was synthesized from 2 μg DNase-treated total RNA using *amfivert Platinum* cDNA Synthesis Master Mix (GenDEPOT Inc., TX, USA). All reactions were performed in triplicate using the SYBR Premix Ex Taq (Takara Bio, Inc., Otsu, Shiga, Japan) and carried out in an Applied Biosystems StepOnePlus Real-Time PCR System (Applied Biosystems Inc., Foster, CA, USA) according to the manufacturers’ instructions. The reaction cycle was: 1 cycle of 95°C for 30 s followed by 40 cycles of 95°C for 5 s and 60°C for 34 s. The expression level of *OsActin1* was used to normalize the results.

### Accession codes

The resequencing data has been deposited in EMBL-EBI (http://www.ebi.ac.uk) under the accession numbers: ERP008697 (*Dongjin*), ERP008700 (*Jado*), ERP008712 (*Hwangdo*), and ERP008713-ERP008719 (seven mutant lines). In addition, the RNA-seq data set has been deposited in EMBL-EBI with accession numbers from ERP008763 to ERP008813.

## Results and Discussion

### Genetic variation among the genomes

To investigate the genetic variation underlying leaf color in rice plants, we identified the sequence variations between each of the 10 accessions and the rice reference sequence (*Nipponbare*). Then, we investigated the variations specific to each accession, to understand the genetic basis of the phenotypic variation in leaf color. The sequencing results yielded a total 1,814 × 10^6^ reads, and the mapped reads covered 96.9% of the *Nipponbare* reference genome, which contained 373.9 Mbp. The mapping ratio, which is the portion of reads that could be uniquely mapped onto the reference genome, for the different accessions varied from 97% to 99%. The final effective mapping reads depth ranged from 34.5× to 42.9×, with an average of 38.9× across all the genomes. Finally, after considering only the positions for which more than three reads were mapped to the same site, the mapping coverage of the reference genome was 95.2%. Table 1 shows the results of the reference-genome assemblies for the 10 rice leaf-color accessions. The mapping percentage of the total contigs is shown with the statistics for the unmapped reads from each of the 10 rice accessions.

**Table 1:** Reference genome assemblies of 10 leaf-color rice accessions onto reference genome.

Sample	Number of reads	Mapped reads			All mapped(bp)		3+mapped(bp)^a^	Coverage
		Number	Coverage	Depth	Size(bp)	Coverage		
D052	185,047,892	147,984,209	99.5%	39.04	364,932,999	97.6%	360,269,782	96.4%
D056	190,240,726	155,463,792	99.5%	41.02	364,928,017	97.6%	359,714,657	96.2%
D101	189,411,406	154,914,741	99.4%	40.86	365,135,240	97.7%	360,571,370	96.4%
D120	177,906,528	143,375,391	99.5%	37.85	364,970,666	97.6%	360,416,405	96.4%
D122	202,236,270	162,690,260	99.4%	42.94	363,861,008	97.4%	357,142,960	95.5%
D128	194,082,738	156,793,587	99.3%	41.38	364,940,501	97.6%	360,174,165	96.3%
D131	176,233,928	145,215,869	99.4%	38.36	364,151,236	97.4%	358,394,847	95.9%
Hwangdo	166,716,980	132,359,524	97.9%	34.55	342,025,104	91.5%	329,787,805	88.2%
Jado	167,790,976	363,379,096	97.2%	36.48	363,379,096	97.2%	354,890,848	94.9%
Dongjin	164,390,288	136,409,677	99.5%	36.03	364,784,522	97.6%	358,413,928	95.9%
Average	181,405,773	169,858,615	99.1%	38.9	362,310,839	96.9%	355,977,677	95.2%

3+mapped(bp)^a^: base pair of nucleotide which mapped over 3 reads on one site.

The *Jado* cultivar with six contigs had the highest minimum N50 value (2,882 bp) among the 10 rice accessions (S1 Table). We identified gene losses and gains based on genetic differences separating the rice accessions using multiple genome alignment. Those differences include the effects of recombination, which can create a mosaic pattern of homology even among closely related leaf-color accessions. We identified averages of 195 gene losses and 92 gene gains among the 10 rice accessions (Table 2), and we detected 33 transposable elements, which were associated only with gene losses.

**Table 2:** The statistics of gene loss and gain events from 10 accessions.

		D052	D056	D101	D120	D122	D128	D131	Hwangdo	Jado	Dongjin	Average
Loss genes		167	147	98	155	244	104	148	270	395	224	195
	characterized genes	29	31	19	27	52	23	33	61	102	43	42
	unknown genes	105	95	62	96	148	65	88	169	233	137	120
	transposable element	33	21	17	32	44	16	27	40	60	44	33
Gain genes		79	70	85	78	74	81	82	317	2	54	92
	characterized genes	26	20	25	25	21	20	23	79	0	16	25
	unknown genes	53	50	60	53	53	61	59	238	2	38	67

We identified 3,880,945 unique SNPs among the 10 rice accessions and an average of 713,114 SNPs per accession. In total, 14.5% of the SNPs were located in CDS regions. Synonymous SNPs made up 49.3% of the total mutations and were more common than non-synonymous SNPs. The percentage of total SNPs appearing in intronic regions ranged from 15.9% to 16.5% with a mean of 16.3%. The numbers of SNPs and small InDels were similar among all the rice accessions, except for the *Hwangdo* cultivar, which had a very different genetic background and contained approximately 10 times more mutations than the other accessions (Table 3).

**Table 3:** Distribution of SNPs within various genetic region from 10 rice accessions.

Cultivar	Total	CDS				Intron		UTR				Promoter		InterRegion	
		SY^a^	NS^b^	Total				5'-UTR		3'-UTR					
D052	336,101	36,538	20,701	57,239	17.0%	55,479	16.5%	3,126	0.9%	5,561	1.7%	70,529	21.0%	144,167	42.9%
D056	334,957	36,227	20,033	56,260	16.8%	54,989	16.4%	3,031	0.9%	5,669	1.7%	71,375	21.3%	143,633	42.9%
D101	563,811	53,244	30,630	83,874	14.9%	92,100	16.3%	5,567	1.0%	10,933	1.9%	129,283	22.9%	242,054	42.9%
D120	334,278	36,596	20,208	56,804	17.0%	55,119	16.5%	3,071	0.9%	5,590	1.7%	70,520	21.1%	143,174	42.8%
D122	382,402	37,808	22,855	60,663	15.9%	62,022	16.2%	2,900	0.8%	5,891	1.5%	83,085	21.7%	167,841	43.9%
D128	415,239	44,937	26,523	71,460	17.2%	66,203	15.9%	3,468	0.8%	6,426	1.5%	86,776	20.9%	180,906	43.6%
D131	421,219	45,124	26,040	71,164	16.9%	68,145	16.2%	3,246	0.8%	6,558	1.6%	90,200	21.4%	181,906	43.2%
Hwangdo	3,724,388	266,772	212,176	478,948	12.9%	612,986	16.5%	29,788	0.8%	70,171	1.9%	894,150	24.0%	1,638,345	44.0%
Jado	294,871	30,632	16,895	47,527	16.1%	48,323	16.4%	2,548	0.9%	4,926	1.7%	63,758	21.6%	127,789	43.3%
Dongjin	323,873	31,295	18,594	49,889	15.4%	52,207	16.1%	2,966	0.9%	6,364	2.0%	72,963	22.5%	139,484	43.1%
Average	713,114	61,917	41,466	103,383	14.5%	116,757	16.4%	5,971	0.8%	12,809	1.8%	163,264	22.9%	310,930	43.6%

SY^a^: synonymous SNPs in CDS region, NS^b^: non-synonymous SNPs in CDS region.

In order to reveal the SNP distributions and gap densities, we mapped the SNPs from each of the 10 rice accessions onto the genomic sequences of the reference genome. The SNP density was based on the distribution of SNPs per 10-kb interval, and the presence of gaps was based on regions with a mapped reads depth of 0–4× despite being flanked on both sides by regions of at least 1 kb with reads depth greater than 20×. The SNP distributions among the seven mutant lines of the *Dongjin* cultivar were similar, and the SNPs in specific locations were mainly retrotransposon genes. The *Hwangdo* cultivar had the highest SNP density, which we assumed to be the cause of 10-times-greater number of SNPs in that cultivar compared with the other accessions. S2 Fig shows the variation among all the SNP distributions per 10-kb interval and the gap densities for rice chromosome 4.

### Population structure and gene-expression analysis

**Fig 1:**
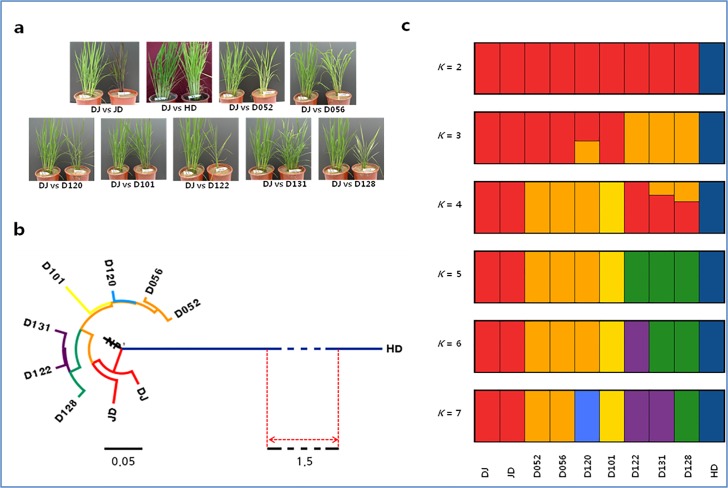
Population structure of 10 rice leaf-color accessions. (a) Comparison of the phenotypes of nine leaf-color accessions with that of the *Dongjin* cultivar (wild type). (b) A neighbor-joining phylogenetic tree of the rice genomes based on 3,880,945 high-quality SNPs, which were identified in all 10 of the rice accessions. The phylogenetic tree was generated with 1,000 bootstrap repetitions, and all nodes were clustered with bootstrap values (greater than 95%) except for the *Hwangdo*-cultivar node. (c) A population-structure analysis using FRAPPE with high-quality SNPs. Each accession is represented by a vertical bar.

To investigate population structure using the 3,880,945 total SNPs among the 10 rice accessions, we estimated the ancestry and admixture proportion of each accession assuming that K populations exist based on a maximum likelihood method using the FRAPPE program. Ancestry was analyzed by increasing K (i.e., the number of populations) from 2 to 7. In addition, the phylogenetic-tree analysis using PHYLIP and FigTree indicated a cluster of high relatedness among the *D052*, *D056*, and *D120* mutant lines. Another composite cluster indicated homologous relationships among the *D122*, *D131*, and *D128* mutant lines. The *Hwangdo* cultivar had the greatest distance from the other nine accessions, which tended to cluster separately on the phylogenetic tree (Fig 1).

**Fig 2:**
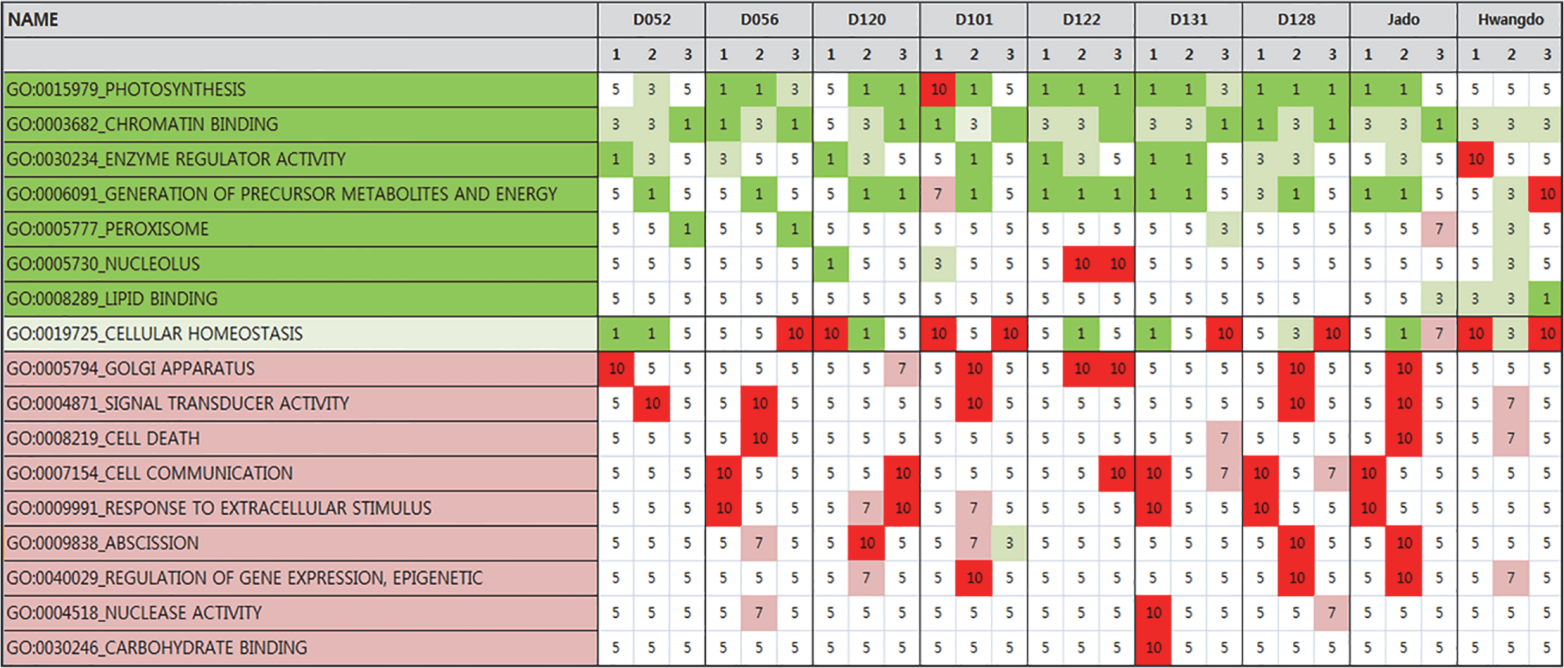
Genome-wide expression profiles from the RNA-seq experiments with 10 rice accessions. Expression values were generated by the GSEA method using FPKM values. The expression of photosynthesis-related genes (GO:0015979) was decreased in all the rice accessions. The GO categories were selected by p-value (≤ 0.05) cutoff, and green and red colors represent down-regulated and up-regulated categories, respectively.

In order to interpret the genome-wide expression profiles for genes related to photosynthesis and for cellular-metabolism genes playing an important role in leaf-color characterization, we used the GSEA method and GO analysis with the FPKM values from the RNA-seq experiments. The expression values for the *Hwngdo* and *Jado* cultivars and the *Dongjin*-derived mutant lines were assessed relative to the expression values of the *Dongjin* cultivar. The genes related to photosynthesis were enriched with down-regulated genes, and the genes related to cell homeostasis of external stimulus were enriched with up-regulated genes. Among the GO categories, using p value ≤ 0.05 as a cutoff for significance, the expression levels of photosynthesis-related genes were reduced, except for those in the *Hwangdo* cultivar, and the expression levels of cellular homeostasis-related genes were increased in all of the rice accessions (Fig 2). The expression results were assumed to be caused by increases in the expression of cell-communication genes for recovery from growth under irregular conditions and decreases in photosynthesis efficiency due to genetic variation in the cell process metabolism.

### Genes potentially associated with leaf-color variation

We used Pathway Studio (v 9.0, Ariadne Genomics, Inc., Rockville, MD, USA) to identify candidate genes for leaf color-related metabolism or biosynthesis. 'Transporter', 'leaf color', 'secondary metabolite', and ' chlorophyll' were used as keywords. The proteins thus identified were entered into Pathway Studio in order to determine the relevant pathways. Each protein was confirmed by the PubMed Medline (http://www.ncbi.nlm.nih.gov/pubmed/) hyperlink embedded in each node and KEGG pathway. We identified 375 candidate genes based on direct involvement in the relevant networks. In addition, we run a Fisher's exact test analysis with the p-value cut off at 0.05 to identify pathways enriched in the differentially expressed gene set for significant up-regulation or down-regulation (i.e., greater than 2.0-fold changes in expression levels) among three developmental stages. With the Elsevier pathways database of Pathway Studio, when the proteins in the pathway are listed by the FPKM expression levels of specific genes, the pathway nodes selected. The total selected pathway nodes of the protein group linked the related genes among the differentially expressed gene set table. Total 2,317 candidate genes were identified based on evidence found in the literature. To identify genes for specific pathways among the 2,692 genes including 375 genes, we screened the final candidate genes using same process with Fisher's exact test (P ≤ 0.05). Finally, we identified 47 candidate genes for further analysis for potential involvement in leaf-color biosynthesis and/or metabolism in ornamental rice plants. The candidate genes fell into two groups: those related to macronutrient (i.e., magnesium and sulfur) transport and those related to flavonoid pathways (e.g., anthocyanidin biosynthesis). According to our analysis, the seven leaf-color accessions (*D052*, *D056*, *D101*, *D120*, *D122*, *D128*, and *D131*) generated from the *Dongjin* cultivar were mainly distinguished by breaks in the macronutrient-transporter pathways, and the three leaf-color cultivars (*Hwangdo*, *Jado*, and *Dongjin*) were mainly distinguished by differences in gene expression rather than DNA variation.

### Genes involved in magnesium and sulfate transport

**Fig 3:**
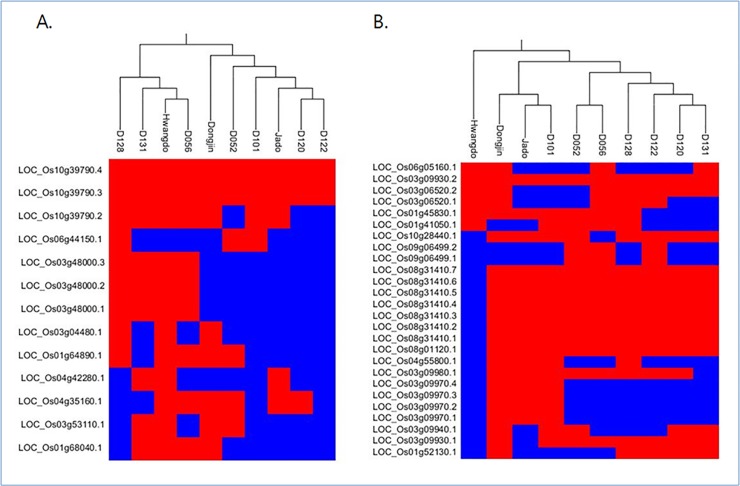
Hierarchical clustering of magnesium-transporter and sulfur-transporter genes among 10 rice accessions. For each rice accession, the reads were mapped to the reference genome, and the coding sequences of magnesium-transporter and sulfur-transporter genes were isolated. The red and blue colors represent normal or broken status (which induced a gap or frame shift in the CDS region), respectively. (a) Phylogenetic relationships among 13 anchored genes identified as magnesium transporters in the rice genome. (b) Phylogenetic relationships among 26 anchored genes identified as sulfur transporters in the rice genome.

The rice genome is reported to have nine magnesium transporters (*CorA*) [25]. We analyzed the genetic variation among the magnesium-transport genes using 13 transcripts from nine *CorA* homologs in the 10 rice accessions. The variation in the magnesium transporter MRS2/LPE10 (IPR026573) was mainly due to deletions. The *D120* and *D122* mutant lines had deletions in 10 and 11 of the 13 transcripts, respectively (S2 Table). Those cultivars show the symptoms of magnesium deficiency, which gives the leaves a marbled appearance of chlorosis and leads to premature aging of the plant. The rice genome is reported to have 14 sulfate-transporter genes [11]. We analyzed the genetic variation in the sulfate-transporter genes using 26 transcripts of 14 genes from the 10 rice accessions. The *Hwangdo* cultivar, which has the most brightly colored leaves among the 10 rice accessions, had changes in 20 different transcripts of 10 genes (S3 Table). The *LOC_Os03g09970* transcript had the most genetic variation among the 10 accessions relative to the reference genome. Using the ClustalW program, we performed a multiple sequence alignment around the *LOC_Os03g09970* transcript region on the reference genome. A specific coding SNP was detected, in which glycine (GGC/GGU) was changed to serine (AGC/AGU) because of an allele change (G → A) on chromosome 3. That SNP was detected in the *D052*, *D056*, *D120*, *D122*, *D128*, and *D131* mutant lines; the *Hwangdo* cultivar did not encode any amino acid at the same location because of a gap. *Dongjin*, *Jado*, and *D101*, which are not bright-leaf varieties, were homozygous for the 'G' allele at the same position (S3 Fig). The glycine-to-serine change replaces a relatively small amino acid (glycine) with a larger amino acid (serine). Alterations of serine residues, which are essential for phosphorylation reactions, can cause the hyperphosphorylation of sulfate transporters, inhibiting sulfate uptake [26,27]. The glycine-to-serine change appeared in the accessions that commonly show the markedly bright leaves and marbled appearance of chlorosis, suggesting that the bright-leaf phenotype is caused by changes in membrane-transporter activity due to an amino acid change to the more complex serine from the simpler glycine. Hierarchical clustering was performed to define similarities among the rice accessions based on the disruption of genes involved in the transport of both magnesium and sulfur. In order to identify the disrupted genes, we isolated the coding sequences of genes involved in magnesium and sulfur transport and mapped the sequencing reads to those genes in the reference genome. The clustering showed many disrupted magnesium-transport genes in the *D120* and *D122* mutant lines (Fig 3A) and disrupted sulfur-transport genes in the *Hwangdo* cultivar (Fig 3B).

**Fig 4:**
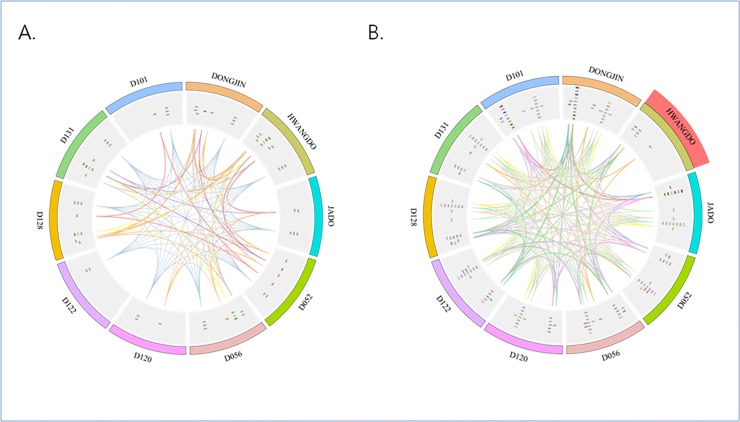
Circos diagram of the expression of genes related to magnesium transport and sulfur transport. The homologous genes in each group are plotted against nine other rice accessions as their counterparts. Individual magnesium-transporter and sulfur-transporter groups are shown as a similarity index representing the shared genes among the genomes. (a) Circos diagram of magnesium transporters. (b) Circos diagram of sulfur transporters. The *Hwangdo* cultivar had gene disruptions in the genes for sulfur-transporter biosynthesis.

In order to verify the results of the hierarchical clustering, we compared the clustering results to the genomic variation and FPKM data from the RNA-seq experiments. The hierarchical clustering was performed using five rice accessions that were assumed to have alterations in sulfur transporters based on our previous analysis. Using 15 FPKM values from the RNA-seq experiments performed at three different stages of leaf development for the five cultivars, the clustering of the sulfate-transporter genes showed that the expression patterns reflected the genomic variation (S4 Fig). The Circos diagram shows the relative positions of the rice accessions and both transporter genes that were identified by our hierarchical clustering analysis. In the sulfur-transporter diagram, the gene disruptions in the *Hwangdo* cultivar reflect the results of the hierarchical clustering (Fig 4).

### Genes involved in the anthocyanin pathway

The genetic characterization of the anthocyanin pathway in relation to leaf-color variation mainly showed variation in the binding of transcription factors rather than sequence changes in the CDS of the genes. Therefore, we assumed that the phenotypic variation in pigments is mainly caused by differences in gene expression. According to the Kyoto Encyclopedia of Genes and Genomes (KEGG, http://www.genome.jp/kegg/) database, the flavonoid pathway is the key pathway for anthocyanin production among the multiple plant secondary-metabolite pathways (KEGG, map00941). The most effective mechanism for increasing anthocyanine production is to increase the anthocyanin precursor and decrease the reductase. The anthocyanidins (pelargonidin, cyanidin, and delphinidin) are the precursors of anthocyanin, and their conversion to anthocyanin requires two enzymes: EC 1.1.1.219 and EC 1.14.11.19 [28,29]. High anthocyanin-reductase activity (EC 1.17.1.3 and EC 1.3.1.77) obstructs anthocyanin production [30,31]. Based on the 30 FPKM values of the 10 rice accessions at three developmental stages, three previously reported precursor genes for anthocyanin production (anthocyanidin 3-o-glucosyltransferase: *LOC_Os07g32020*, dihydroflavonol reductase: *LOC_Os 01g44260*, and leucoanthocyanidin dioxygenase: *LOC_Os01g27490*) were strongly up-regulated in the *Jado* cultivar. The expression of anthocyanin reductase (i.e., leucoanthocyanidin reductase: *LOC_Os03g15360* and anthocyanidin reductase: *LOC_Os04g53850* and *LOC_Os04g53920*) showed little change among the 10 rice accessions (S4 Table).

**Fig 5:**
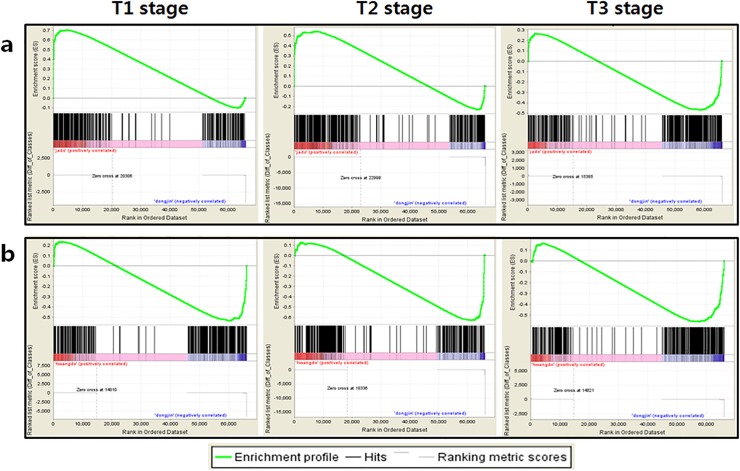
Gene-expression profiling for anthocyanin biosynthesis using gene-set enrichment analysis (GSEA) score curves. Examples of enrichment plots are shown for categories identified using the GSEA method at three different stages of leaf development (germination + 30 days, + 45 days, and + 60 days) between the (a) *Jado* and (b) *Hwangdo* cultivars. Black bars represent the positions of members of the category in the ranked list together with the running enrichment score (plotted in green).

In addition, the *Jado* and *Hwangdo* cultivars, which were assumed to have altered anthocyanin biosynthesis based on our previous analysis, were analyzed to compare them with the *Dongjin* cultivar using the GSEA method. First, we distinguished two groups, the “up group” (S5 Fig, red box) and the “down group” (S5 Fig, blue box), for analyzing the enzyme-expression ratio of the anthocyanin-related genes in the flavonoid pathway. Effective anthocyanine production involves the up-regulation of the up-group genes and the down-regulation of the down-group genes. Second, we applied the GSEA method using the expression of the two groups of genes at three developmental time points using the 30 FPKM values. The expression of the up-group genes was up-regulated in the *Jado* cultivar, except for during the third stage of development, and down-regulated in the *Hwangdo* cultivar. The expression of the down-group genes showed little difference among all the rice accessions (S6 Fig). Third, we performed the same method using only the anthocyanin-pathway genes (KEGG, map00942). Compared with their expression in the *Dongjin* cultivar, the anthocyanin-pathway genes were enriched with up-regulated genes in the *Jado* cultivar and with down-regulated genes in the *Hwangdo* cultivar, similar to the expression patterns of the up group of flavonoid-pathway genes (Fig 5).

We searched the genome variation to determine the cause of the differences in gene expression and found that the anthocyanidin reductase gene (*LOC_Os04g53850* and *LOC_Os04g53920*) had multiple coding SNPs in the *Dongjin* and *Hwangdo* cultivars. The *Jado* cultivar, which has the highest anthocyanin content, showed activation of an NmrA-like family protein at the *LOC_Os04g53850* location due to a partially deleted sequence (S7 Fig). Three anthocyanidin-precursor genes did not have coding SNPs in their coding regions. Therefore, differences in anthocyanin production were assumed to mainly occur due to genomic DNA variation affecting protein modification via the interaction of transcription factors, leading to differences in expression among developmental stages. Also, differences in anthocyanin production were due more to positive regulation than to negative regulation.

### Gene validation using quantitative real-time PCR and transgenic T-DNA insertion mutants

In order to investigate the relationship between leaf-color phenotypes and the selected candidate genes, we used transgenic T-DNA insertion mutants [32] instead of generating new mutant-plant accessions. First, we searched the flanking sequence-tag database of T-DNAs with the gene locus number or location on the chromosome of the 47 candidate genes identified in our analysis. We selected 28 transgenic T-DNA mutants with insertions that matched the targeted gene loci.

**Fig 6:**
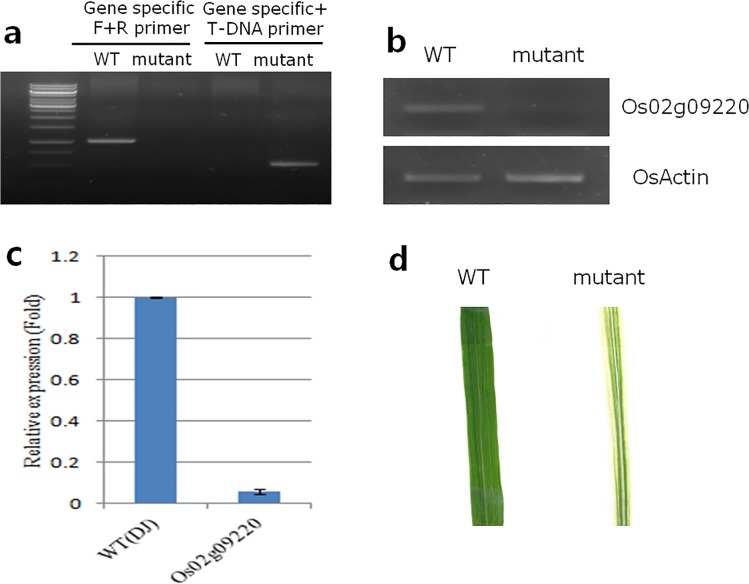
Verification of *Os02g09220* affecting leaf color by qPCR, T-DNA insertion, and phenotypic characterization. (a) PCR of genomic DNA showed the successful generation of the T-DNA insertion mutant. (b) RT-PCR revealed the expression of the *Os02g09220* gene in the mutant. (c) The qRT–PCR analysis of *Os02g09220* expression between the wild-type and T-DNA insertion-mutant plants. (d) Phenotypic comparison of the two plant types.

Second, we planted the seeds of the 28 T-DNA mutants and assessed the leaf-color phenotypes of the resulting plants. Twenty-four of the T-DNA mutant plants did not display the expected leaf-color phenotypes. We performed PCR using the genomic DNA of the four T-DNA mutants that did display the expected phenotypes to verify the correct T-DNA insertions. The specific gene locus was matched to the ‘1B-03717’ T-DNA insertion mutants using the *Os02g09220* gene. This gene, including the *LOC_Os02g09220* transcript, was annotated as a cytochrome P450 family gene by the International Rice Genome Sequencing Project (IRGSP, http://rgp.dna.affrc.go.jp/IRGSP/). /). The results showed that the ‘1B-03717’ T-DNA mutant was successfully generated (Fig 6A); however, the *Os03g46470* (T-DNA ID: 2A-20049), *Os07g13634* (T-DNA ID: 3A-08329), and *Os03g15360* (T-DNA ID: 2D-01256) T-DNA mutants were incompletely generated because of the duplication or deletion of the T-DNA insertion.

Third, we performed reverse-transcriptase PCR (RT-PCR) to reveal the expression of the gene in the mutant, and qPCR showed the difference in gene expression between the wild-type cultivar and the mutant (Fig 6B and 6C). Fourth, we investigated the sequence variation of the *LOC_Os02g09220* transcript among the 10 rice accessions. The accessions showing leaf chlorosis were affected more by gaps on the gene locus than by allelic variation. The *D128* and *D122* mutant lines, which show the most leaf chlorosis, had a gap of 154 and 268 bp, respectively (S5 Table).

Finally, the phenotypic results showed that the ‘1B-03717’ T-DNA mutant had greater leaf chlorosis compared with the wild-type plants (Fig 6D). We assumed that the change in leaf chlorosis was the result of the *LOC_Os02g09220* transcript having decreased expression due to the gene-loss event.

In addition, the *LOC_Os01g16040* transcript was reported previously to encode the 3’-Phosphoadenosine 5’-Phosphosulfate Transporter1 (PAPST1) protein, which plays a role in chloroplast retrograde signaling and development, resulting in leaf chlorosis at an early developmental stage [33]. Although there is no direct evidence linking the functions of PAPST1 to leaf color, our results suggest that that gene plays a role in the regulation of the leaf-color pathway. Taken together, the results obtained from the T-DNA insertion mutant validate our complex screening method using transgenic T-DNA mutants to identify functional genes by combining genomic-variation and gene-expression analyses.

## Conclusions

We resequenced a total of 10 rice leaf-color accessions to approximately 40× depth and >95% coverage using the Illumina Hiseq 2000 platforms, and we performed 30 RNA-seq experiments that compared gene expression at three different developmental stages. Using an integrated analysis of the RNA-seq and whole-genome resequencing data, we were able to obtain a unique view of the genetic expression patterns, transcript structure, and population-level transcriptome differences among leaf-color accessions and standard cultivars of rice. The leaf color-related genes that we identified were characterized into two groups, which included genes related to macronutrient (i.e., magnesium, and sulfur) transport and genes related to flavonoid pathways, including anthocyanidin biosynthesis. To verify the candidate genes identified by our analyses, we performed qPCR using transgenic T-DNA insertion mutants. Although the identified genes require additional validation to further confirm the whole-genome resequencing and RNA-seq experimental strategy, our study demonstrates the potential of our screening method combining DNA-variation data and RNA-seq transcriptome data to isolate genes involved in complex biosynthetic networks and pathways.

## Supporting Information

S1 Fig A leaf color-phenotype image of the 10 rice accessions. (TIF)

S2 Fig Distribution of the variation events on chromosome 4 of the 10 rice leaf-color accessions. (TIF)

S3 Fig Visualization of the multiple sequence alignment around the *LOC_Os03g09970* gene in the 10 accessions relative to the reference genome. (TIF)

S4 Fig Hierarchical clustering of the sulfur-transporter genes with 15 FPKM values from five rice accessions. (TIF)

S5 Fig The map of the flavonoid-biosynthesis pathway (KEGG, map00941), which serves a multitude of functions including anthocyanin biosynthesis. (TIF)

S6 Fig Gene-expression profiling for flavonoid biosynthesis using score curves of the GSEA method. (TIF)

S7 Fig Sequence variation within partial anthocyanidin reductase as *LOC_Os04g53850* and *LOC_Os04g53920*. (TIF)

S1 Table The statistics of *de novo* contigs assembled from unmapped reads from each rice accession. (PDF)

S2 Table Transcript variation of magnesium transporter-related genes. (PDF)

S3 Table Transcript variation of sulfur transporter-related genes. (PDF)

S4 Table Expression values of anthocyanin biosynthesis-related genes at three developmental stages in the 10 rice accessions. (PDF)

S5 Table The statistics of gap the information from two transcripts, *LOC_Os02g09220* and *LOC_Os01g16040*. (PDF)
