# Supplementary figures and images for: Whole-Genome Resequencing and Transcriptomic Analysis to Identify Genes Involved in Leaf-Color Diversity in Ornamental Rice Plants
Source: PLoS One. 2015 Apr 21;10(4):e0124071. doi: 10.1371/journal.pone.0124071 (PMC4405343; doi:10.1371/journal.pone.0124071)

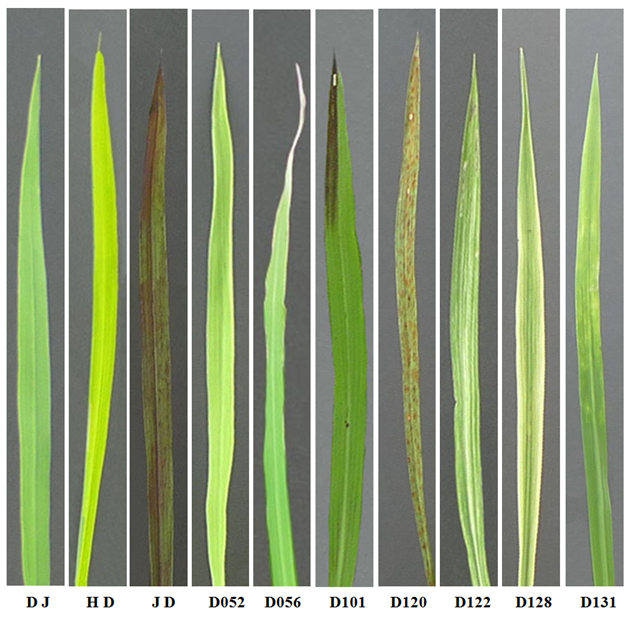

Supplement: S1 Fig — (TIF) [file pone.0124071.s001.tif]

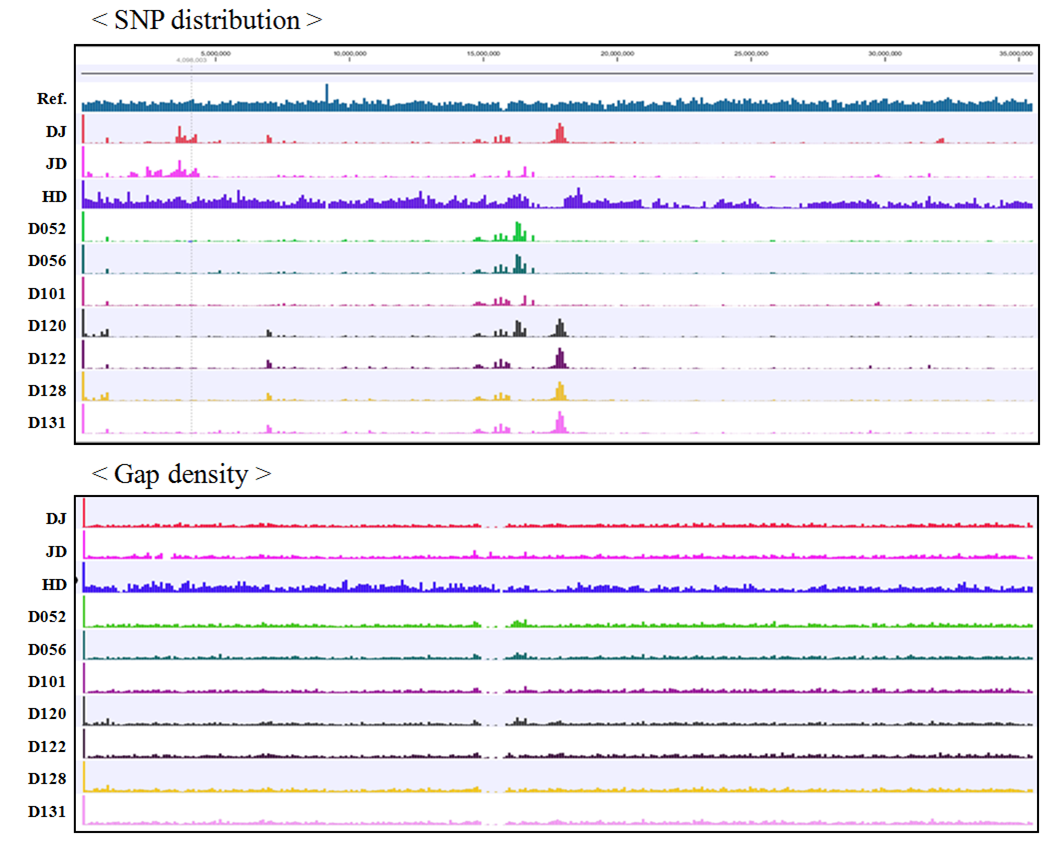

Supplement: S2 Fig — (TIF) [file pone.0124071.s002.tif]

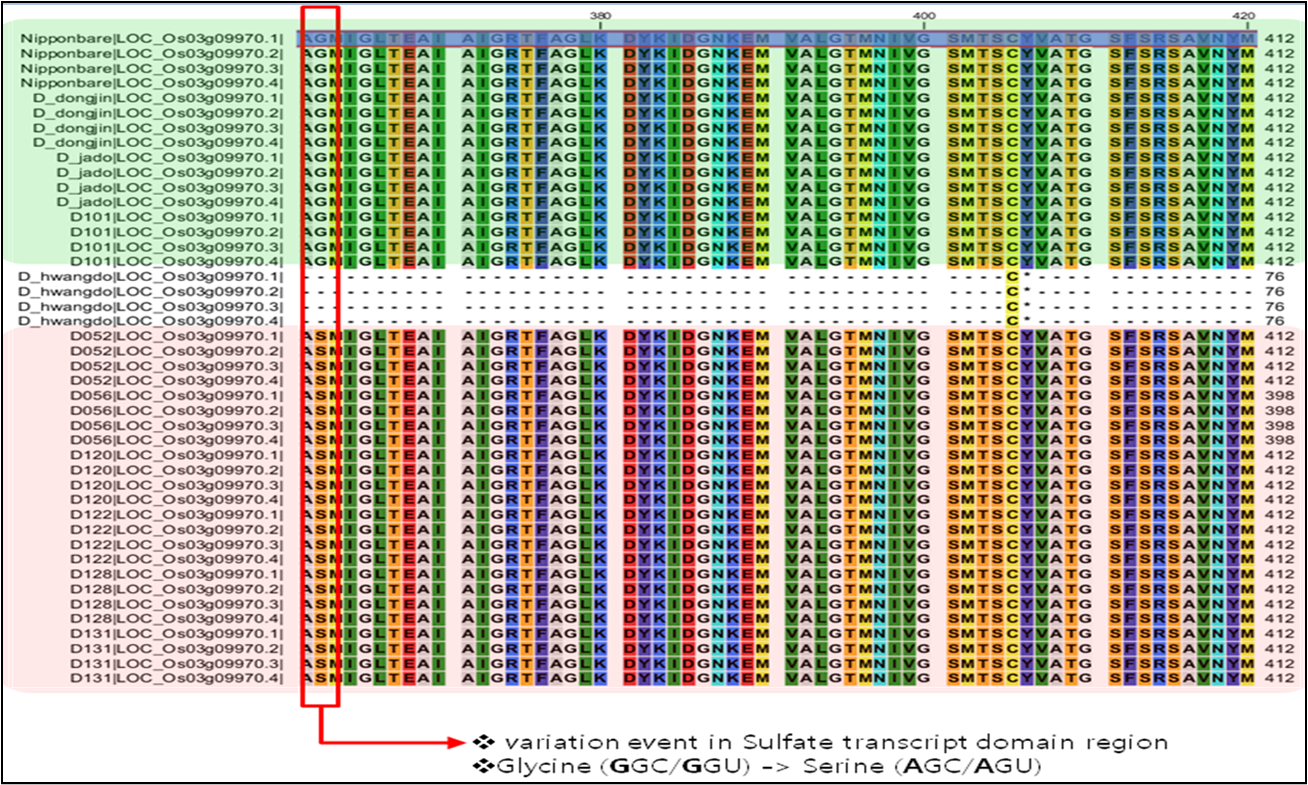

Supplement: S3 Fig — (TIF) [file pone.0124071.s003.tif]

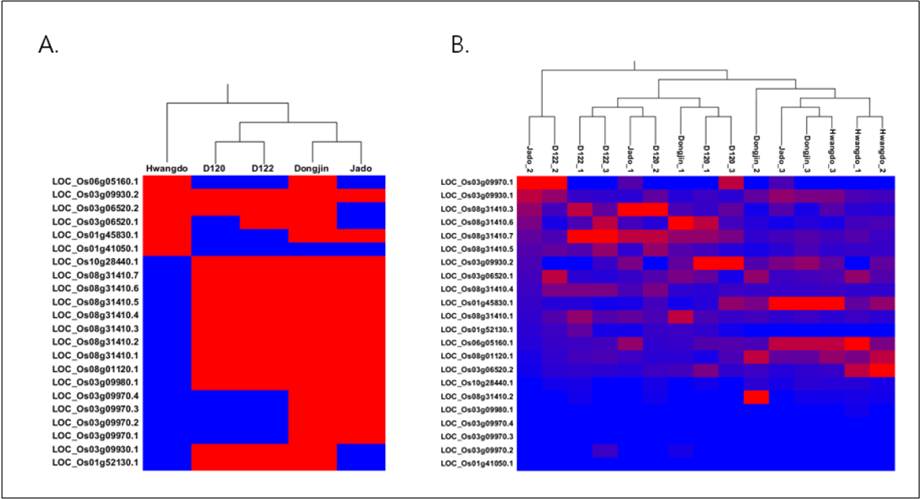

Supplement: S4 Fig — (TIF) [file pone.0124071.s004.tif]

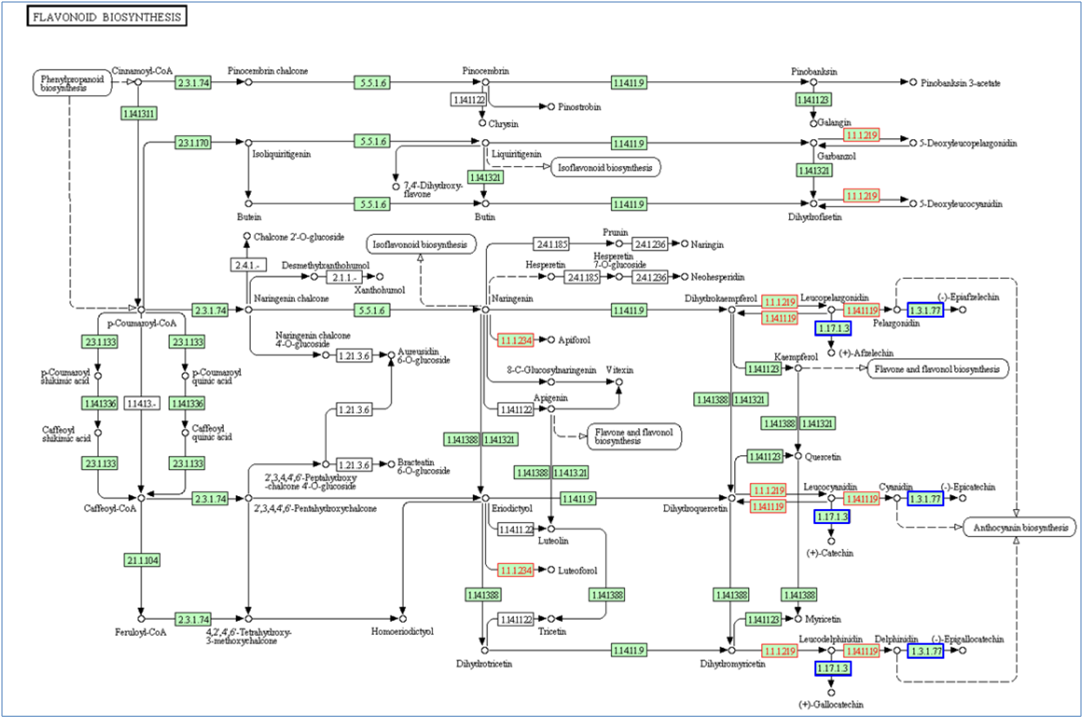

Supplement: S5 Fig — (TIF) [file pone.0124071.s005.tif]

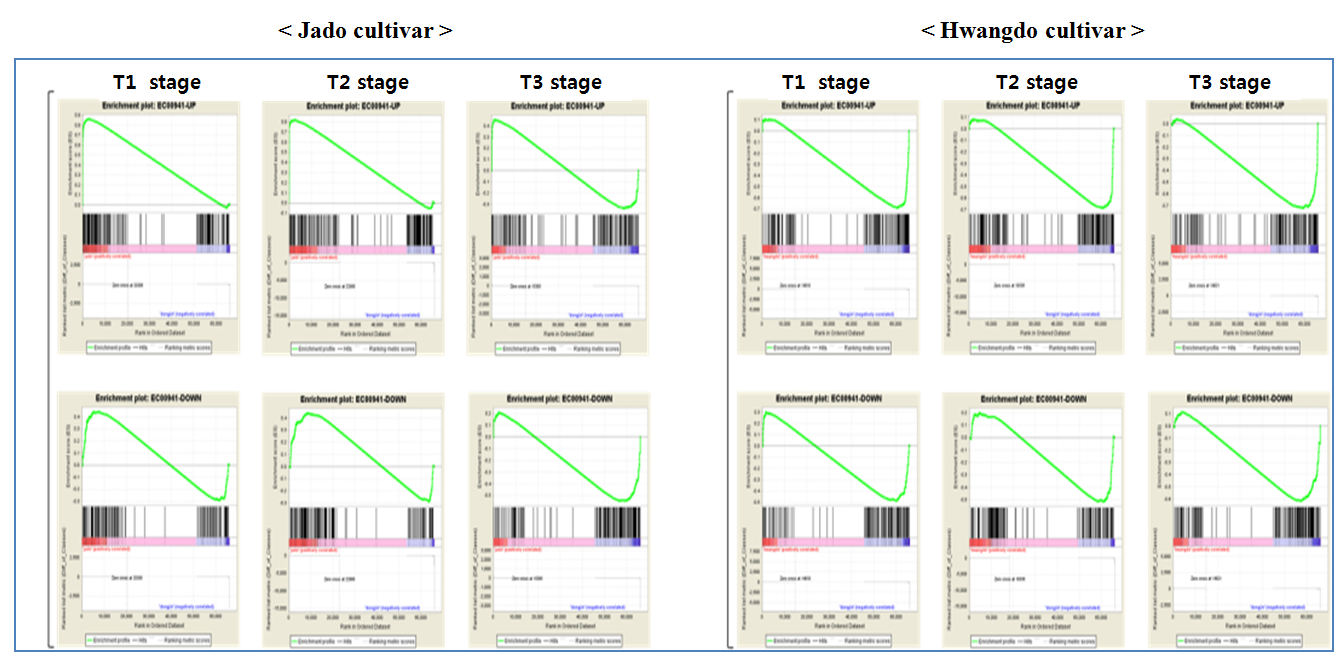

Supplement: S6 Fig — (TIF) [file pone.0124071.s006.tif]

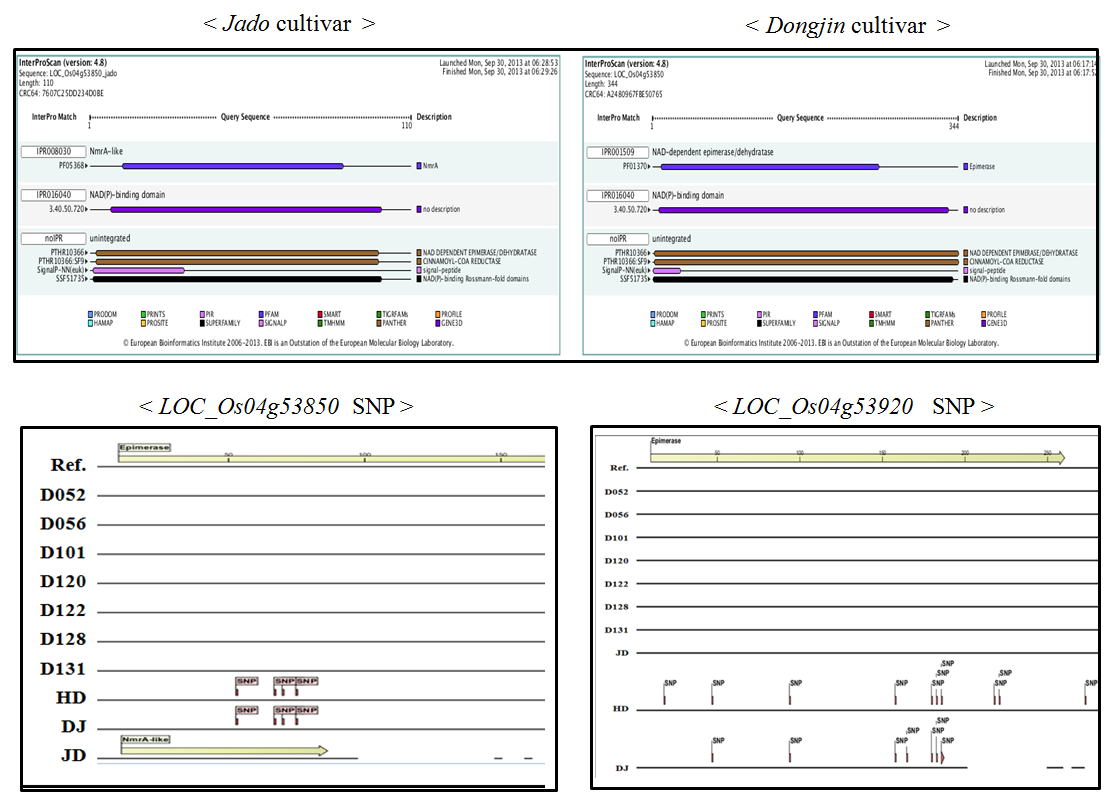

Supplement: S7 Fig — (TIF) [file pone.0124071.s007.tif]
