# Supplementary material for: Whole-Genome Resequencing and Transcriptomic Analysis to Identify Genes Involved in Leaf-Color Diversity in Ornamental Rice Plants
Source: PLoS One. 2015 Apr 21;10(4):e0124071. doi: 10.1371/journal.pone.0124071 (PMC4405343; doi:10.1371/journal.pone.0124071)
Supplement: S1 Table — (PDF) [file pone.0124071.s008.pdf]

Table S1. The statistics of de novo contig which was assembled with unmapped reads from each accession.

	D052	D056	D101	D120	D122	D128	D131	Hwangdo	Jado	Dongjin	Average
No. contigs	400	367	458	402	386	473	445	1,814	6	329	508
Total length, bp	1,551,592	1,426,929	1,773,112	1,555,609	1,497,981	1,765,671	1,724,289	6,746,840	20,476	1,307,593	1,937,009
N50, bp	4,147	4,115	4,079	4,178	4,104	4,000	4,134	3,891	2,882	4,200	3,973
Max, bp	14,106	19,299	22,370	14,039	14,854	14,705	14,069	18,065	4,774	16,855	15,314
Min, bp	2,007	2,005	2,005	2,000	2,004	2,000	2,000	2,000	2,582	2,000	2,060
