# Supplementary material for: Whole-Genome Resequencing and Transcriptomic Analysis to Identify Genes Involved in Leaf-Color Diversity in Ornamental Rice Plants
Source: PLoS One. 2015 Apr 21;10(4):e0124071. doi: 10.1371/journal.pone.0124071 (PMC4405343; doi:10.1371/journal.pone.0124071)
Supplement: S2 Table — (PDF) [file pone.0124071.s009.pdf]

Table S2. Transcript variation of magnesium transporter related genes.

Transcript	Gene	D052	D056	D101	D120	D122	D128	D131	Hwangdo	Jado	Dongjin
LOC_Os01g64890.1	Os01g0869200	-	-	×	×	×	-	×	-	×	-
LOC_Os01g68040.1	Os01g0908500	×	-	×	×	×	×	-	-	×	-
LOC_Os03g04480.1	Os03g0137700	×	×	×	×	×	-	×	-	×	-
LOC_Os03g48000.1	Os03g0684400	×	-	×	×	×	-	-	-	×	×
LOC_Os03g48000.2	〃	×	-	×	×	×	-	-	-	×	×
LOC_Os03g48000.3	〃	×	-	×	×	×	-	-	-	×	×
LOC_Os03g53110.1	Os03g0742400	-	×	×	×	×	×	-	-	×	-
LOC_Os04g35160.1	Os04g0430900	-	-	×	-	×	×	×	-	-	-
LOC_Os04g42280.1	Os04g0501100	×	×	×	×	×	×	-	-	-	×
LOC_Os06g44150.1	Os06g0650800	-	×	-	×	×	-	×	×	×	×
LOC_Os10g39790.2	Os10g0545000	×	-	-	×	×	-	-	-	-	-
LOC_Os10g39790.3	〃	-	-	-	-	-	-	-	-	-	-
LOC_Os10g39790.4	〃	-	-	-	-	-	-	-	-	-	-
Total (transcript/gene)		6/4	9/5	4/2	3/1	2/0	9/5	9/5	12/8	5/3	8/6

"-" symbol (normal in the CDS region), "×" symbol (broken in the CDS region)
