# Supplementary material for: Whole-Genome Resequencing and Transcriptomic Analysis to Identify Genes Involved in Leaf-Color Diversity in Ornamental Rice Plants
Source: PLoS One. 2015 Apr 21;10(4):e0124071. doi: 10.1371/journal.pone.0124071 (PMC4405343; doi:10.1371/journal.pone.0124071)
Supplement: S3 Table — (PDF) [file pone.0124071.s010.pdf]

Table S3. Transcript variation of sulfur transporter related genes.

Transcript	Gene	D052	D056	D101	D120	D122	D128	D131	Hwangdo	Jado	Dongjin
LOC_Os01g41050.1	Os01g0593700	-	-	-	×	×	-	×	-	×	×
LOC_Os01g45830.1	Os01g0645900	-	-	-	×	×	-	×	-	-	-
LOC_Os01g52130.1	Os01g0719300	×	×	×	-	-	-	-	×	×	-
LOC_Os03g06520.1	Os03g0161200	×	-	×	×	-	-	×	-	×	-
LOC_Os03g06520.2	〃	×	-	×	-	-	-	-	-	×	-
LOC_Os03g09930.1	Os03g0195300	-	-	-	-	-	-	-	×	×	-
LOC_Os03g09930.2	〃	-	-	-	-	-	-	-	-	-	-
LOC_Os03g09940.1	Os03g0195450	-	×	-	-	×	×	-	×	×	-
LOC_Os03g09970.1	Os03g0195800	×	×	-	×	×	×	×	×	-	-
LOC_Os03g09970.2	〃	×	×	-	×	×	×	×	×	-	-
LOC_Os03g09970.3	〃	×	×	-	×	×	×	×	×	-	-
LOC_Os03g09970.4	〃	×	×	-	×	×	×	×	×	-	-
LOC_Os03g09980.1	Os03g0196000	-	-	-	-	-	-	×	×	-	-
LOC_Os04g55800.1	Os04g0652400	×	×	-	×	×	-	×	×	-	-
LOC_Os06g05160.1	Os06g0143700	×	-	×	×	×	×	-	-	×	-
LOC_Os08g01120.1	Os08g0101500	-	-	-	-	-	-	-	×	-	-
LOC_Os08g31410.1	Os08g0406150	-	-	-	-	-	-	-	×	-	-
LOC_Os08g31410.2	〃	-	-	-	-	-	-	-	×	-	-
LOC_Os08g31410.3	〃	-	-	-	-	-	-	-	×	-	-
LOC_Os08g31410.4	〃	-	-	-	-	-	-	-	×	-	-
LOC_Os08g31410.5	〃	-	-	-	-	-	-	-	×	-	-
LOC_Os08g31410.6	〃	-	-	-	-	-	-	-	×	-	-
LOC_Os08g31410.7	〃	-	-	-	-	-	-	-	×	-	-
LOC_Os09g06499.1	Os09g0240500	-	-	×	×	-	×	×	×	×	×
LOC_Os09g06499.2	〃	-	-	×	×	-	×	×	×	×	×
LOC_Os10g28440.1	Os10g0420400	-	×	-	-	-	-	-	×	-	-
Total (transcript/gene)		17/9	18/9	20/10	15/7	17/8	18/10	15/7	6/4	17/7	23/12

"-" symbol (normal in the CDS region), "×" symbol (broken in the CDS region)
