# Supplementary material for: Whole-Genome Resequencing and Transcriptomic Analysis to Identify Genes Involved in Leaf-Color Diversity in Ornamental Rice Plants
Source: PLoS One. 2015 Apr 21;10(4):e0124071. doi: 10.1371/journal.pone.0124071 (PMC4405343; doi:10.1371/journal.pone.0124071)
Supplement: S4 Table — (PDF) [file pone.0124071.s011.pdf]

Table S4. Expression value of anthocyanin biosynthesis related genes at three developmental stages in the 10 accessions.

Cultivars	L_Os07g32020	L_Os01g44260	L_Os01g27490	L_Os03g15360	L_Os04g53850	L_Os04g53920
Hwangdo_1	0.296	0	0	0	0.499	0
Hwangdo_2	0.327	0	0	0	0.072	0
Hwangdo_3	0.148	0	0	0.094	0.779	0
Jado_1	114.5	308.9	621.0	0	11.662	1.712
Jado_2	162.1	394.2	1180.8	0	0.898	0.7
Jado_3	38.0	72.8	198.2	0	1.945	0.651
Dongjin_1	5.081	0.08	0.058	0	1.511	0
Dongjin_2	4.088	0	0.069	0	0.537	0
Dongjin_3	3.074	0.136	0.275	0	0.789	0
D052_1	36.753	0	0.224	0	0.874	1.111
D052_2	3.007	0.172	0	0	0.337	1.322
D052_3	0.614	0	0.059	0	2.802	0.694
D056_1	3.223	0	0	0	2.54	0.282
D056_2	2.153	0	0.105	0	0.858	0.962
D056_3	0.767	0	0	0	4.01	0.402
D101_1	14.108	123.1	89.3	0	0.474	2.183
D101_2	8.512	62.0	51.9	0	0.503	1.352
D101_3	0.077	0	0	0	0.742	0.525
D120_1	4.952	0	0	0	5.004	0.754
D120_2	7.708	0	0	0.118	2.855	0.486
D120_3	0.807	0	0.057	0	20.886	0.177
D122_1	12.194	0	0.108	0.11	4.999	0.185
D122_2	17.864	0	0.122	0	1.155	0.893
D122_3	6.029	0	0	0.217	11.564	0.094
D128_1	5.156	0	0	0	0.992	0.725
D128_2	3.972	0	0.066	0	0.345	1.793
D128_3	0.809	0	0.106	0	5.807	0.796
D131_1	13.218	0	0	0	1.494	0.42
D131_2	4.366	0	0.061	0	0.741	1.224
D131_3	0.701	0	0	0	2.424	0.752
