# Supplementary material for: Whole-Genome Resequencing and Transcriptomic Analysis to Identify Genes Involved in Leaf-Color Diversity in Ornamental Rice Plants
Source: PLoS One. 2015 Apr 21;10(4):e0124071. doi: 10.1371/journal.pone.0124071 (PMC4405343; doi:10.1371/journal.pone.0124071)
Supplement: S5 Table — (PDF) [file pone.0124071.s012.pdf]

Table S5. The statistics of gap information from two transcripts as *LOC_Os02g09220 and LOC_Os01g16040*.

Transcripts	Cultivars	Chromosome	Gap position		Average depth		Length	Coverage
			Start	End	5'	3'		
LOC_Os02g09220	D052	Chr2	4,740,780	4,740,792	23.65	39.08	13	3.15
	D120	Chr2	4,740,778	4,740,809	21.48	31.94	32	2.66
	D122	Chr2	4,740,346	4,740,538	35.1	24.49	193	3.04
	D122	Chr2	4,740,541	4,740,615	27.21	27.61	75	2.56
	D128	Chr2	4,740,333	4,740,486	38.5	24.05	154	2.24
	D131	Chr2	4,740,420	4,740,444	26.11	20.05	25	4
	D131	Chr2	4,740,450	4,740,454	24.79	20.42	5	4
	D131	Chr2	4,740,484	4,740,486	23.23	21.68	3	4
	D131	Chr2	4,740,503	4,740,539	22.44	23.67	37	3.76
	D131	Chr2	4,740,556	4,740,570	20.45	24.85	15	4
	Jado	Chr2	4,740,546	4,740,594	20.3	20.68	49	2.94
LOC_Os01g16040	D052	Chr1	9,028,411	9,028,601	58.29	21.84	191	1.51
	D052	Chr1	9,028,700	9,028,702	38.68	24.57	3	4
	D120	Chr1	9,028,399	9,028,630	49.44	20.02	232	2.32
